# Clinical response and symptomatic remission in short- and long-term trials of lisdexamfetamine dimesylate in adults with attention-deficit/hyperactivity disorder

**DOI:** 10.1186/1471-244X-13-39

**Published:** 2013-01-29

**Authors:** Greg W Mattingly, Richard H Weisler, Joel Young, Ben Adeyi, Bryan Dirks, Thomas Babcock, Robert Lasser, Brian Scheckner, David W Goodman

**Affiliations:** 1 St Charles Psychiatric Associates/Midwest Research, 4801 Weldon Spring Pkwy, Suite 300, St. Charles, MO, USA; 2 Duke University Medical Center, Durham, NC, and University of North Carolina at Chapel Hill, Chapel Hill, NC, USA; 3 Rochester Center for Behavioral Medicine, Rochester Hills, MI, USA; 4 Shire Development LLC, Wayne, PA, USA; 5 Formerly of Shire Development LLC, Wayne, PA, USA; 6 Johns Hopkins University School of Medicine, Baltimore, MD, USA

**Keywords:** Lisdexamfetamine dimesylate (LDX), Attention-deficit/hyperactivity disorder (ADHD), Adults, Response, Remission

## Abstract

**Background:**

Despite the overall high degree of response to pharmacotherapy, consensus is lacking on how to judge clinical response or define optimal treatment/remission when treating adults with attention-deficit/hyperactivity disorder (ADHD). This study examined clinical response and symptomatic remission in analyses of 2 studies of lisdexamfetamine dimesylate (LDX) in adults with ADHD.

**Methods:**

In a 4-week, double-blind, forced-dose trial, adults with ADHD were randomized to LDX 30, 50, and 70 mg/day (mg/d) or placebo. In a second, open-label, follow-up trial, adults entering from the 4-week study were titrated to an “optimal” LDX dose (30 mg/d [n=44], 50 mg/d [n=112], and 70 mg/d [n=171]) over 4 weeks, and maintained for 11 additional months. The ADHD Rating Scale IV (ADHD-RS-IV) with adult prompts and the Clinical Global Impressions-Improvement (CGI-I) scale assessed efficacy. Clinical response was defined, post hoc, as ≥30% reduction from baseline in ADHD-RS-IV and CGI-I rating of 1 or 2; symptomatic remission was defined as ADHD-RS-IV total score ≤18. Log rank analysis examined overall significance among the treatment groups in time to response or remission.

**Results:**

Four hundred and fourteen participants in the 4-week study and 345 in the open-label, extension study were included in the efficacy populations. All LDX groups improved by ADHD-RS-IV and CGI-I scores in both studies. In the 4-week study (n=414), 69.3% responded and 45.5% achieved remission with LDX (all doses); 37.1% responded and 16.1% achieved remission with placebo; time (95% CI) to median clinical response (all LDX doses) was 15.0 (15.0, 17.0) days and to remission was 31.0 (28.0, 37.0) days (*P*<.0001 overall). In the open-label study, with LDX (all doses), 313 (95.7%) and 278 (85.0%) of 327 participants with evaluable maintenance-phase data met criteria for response and remission, respectively. Of participants who completed dose optimization, 75.2% remained responders and 65.7% remained in remission in the 12-month study. Overall, 285 (82.6%) and 227 (65.8%) of 345 participants were responders and remitters, respectively, at their final visits.

**Conclusion:**

In the long-term study, with open-label, dose-optimized LDX treatment, most adults with ADHD achieved clinical response and/or symptomatic remission; almost two-thirds maintained symptomatic remission over the remaining 11 months.

**Trial registration:**

Clinical Trial Numbers: NCT00334880 and NCT01070394

Clinical Trial Registry: clinicaltrials.gov

**URLs:**

http://www.clinicaltrials.gov/show/NCT00334880

http://www.clinicaltrials.gov/ct2/show/NCT01070394?term=NCT01070394&rank=1

## Background

Attention-deficit/hyperactivity disorder (ADHD) is a common neurobehavioral condition that is estimated to affect 5% to 10% of youths worldwide and approximately 4.4% of adults in the United States [1,2]. Clinical trials of stimulant pharmacotherapy in adults with ADHD indicate high levels of efficacy. Significantly improved ADHD symptom scores (versus placebo) based on randomized, placebo-controlled trials using long-acting formulations of either methylphenidate (MPH) [3,4] or amphetamines have been reported [5,6].

Despite the overall high degree of treatment response, there are very limited guidelines for how to judge clinical response or how to define optimal treatment/remission when treating adults with ADHD [7]. Meta-analyses indicate effect sizes (relative to placebo) of approximately 0.7 with long-acting psychostimulant medications in adults with ADHD [8]. ADHD symptom scores, based on the ADHD Rating Scale IV (ADHD-RS-IV) [9] and other similar scales, as well as effect size estimates have value but do not indicate what percentage of participants may be expected to improve, when improvement may become apparent, and whether improvements persist. Neither do they address whether participants continue to meet diagnostic criteria, particularly criteria related to functional impairments associated with ADHD symptoms.

Few clinical trials have assessed ADHD treatment efficacy in terms of clinical response, and, to date, no trials in adults (to our knowledge) have described rates of symptomatic remission. Throughout the broad field of psychiatry, numerous definitions of “response” have been proposed, all generally aimed at defining individuals who show clinically apparent improvement to a definitive intervention but who still experience some degree of symptoms [10,11]. As reviewed by Steele et al. [12] and based on various clinical trials of participants with ADHD, response to treatment has been operationally defined as improvement from baseline of 25% to 30% in rating scales such as the ADHD-RS-IV or Swanson, Nolan, and Pelham, Version IV (SNAP)-IV. Inherent to this definition of treatment response is that individuals may continue to have symptoms of the disorder because percent reductions do not account for baseline severity levels. For that reason, a definition of clinical response that is both a composite of a percent reduction in symptoms (eg, a 30% improvement in symptom levels from baseline), as well as a second measure of clinical improvement (eg, Clinical Global Impressions-Improvement [CGI-I] [13] of 1 [very much improved] or 2 [much improved]) may be considered a more meaningful measure. Moreover, it has been reported that an approximate 10- to 15-point change or a percent change of approximately 25% to 30% in ADHD-RS-IV scores corresponded to a 1-level change on the CGI-I [14].

Three types of remission have also been proposed for ADHD: syndromatic, symptomatic, and functional [15]. Syndromatic remission is defined as “failing to meet the full diagnostic criteria for ADHD” [15] and was originally described for bipolar disorder by Keck et al. [16] as syndromatic recovery. Symptomatic remission is defined as having fewer than 5 symptoms, the number of symptoms required for a subthreshold ADHD diagnosis [15]. Functional remission is defined as “the loss of partial diagnostic status plus functional recovery” [15]. In clinical practice, this is likely to be thought of as successful treatment, where the participant no longer exhibits the *Diagnostic and Statistical Manual of Mental Disorders, Fourth Edition, Text Revision (DSM-IV-TR)* behavioral diagnostic criteria for ADHD [17,18]. As reviewed by Keck and colleagues, functional recovery indicates that a patient has attained premorbid levels of functioning (eg, work and psychosocial) for a defined extended period of time [16]. Although not assessed in this study, functional outcome measures in conjunction with those of clinical response and symptomatic remission used to evaluate ADHD symptoms may be clinically relevant assessments for managing and treating ADHD.

Researchers have attempted to objectively define clinical response and symptomatic remission using scale-based cutoff thresholds [18,19]. In the Multimodal Treatment Study of Children With ADHD, success or “excellent response” was defined as a SNAP-IV mean per-item score ≤1, indicating symptom ratings of “not at all” to “just a little” and a severity level below the ADHD diagnostic threshold [18]. Similarly, a total score of ≤18 on the ADHD-RS-IV, which scores each of the 18-item *DSM-IV-TR* criteria, defines an ADHD population that is rated on average by the clinician as mildly symptomatic. To define symptoms, each item was rated on a 4-point scale: 0 (never or rarely); 1 (sometimes); 2 (often); and 3 (very often) [9]. On average, a score of 1 (ie, “sometimes”) across the 18-item scale has been proposed as defining symptomatic remission for participants with combined-type ADHD [12]. This cut off score of ≤18 indicates loss of ADHD symptom status such that the clinician considers the individual with ADHD as no longer exhibiting *DSM-IV-TR* symptom criteria [12]. The cut off for symptomatic remission is having clinically minimal (eg, “sometimes ill” on the ADHD-RS-IV and “just a little ill” on SNAP-IV) or no symptoms (eg, “never or rarely” on the ADHD-RS-IV and “not at all” on SNAP-IV), which is considered in the range of a matched control group without ADHD [12].

In the current investigation, rates of clinical response and symptomatic remission were examined based on post hoc analysis of efficacy data from 2 adult clinical trials of lisdexamfetamine dimesylate (LDX) [5,20]. In a short-term (4-week), randomized, placebo-controlled forced-dose escalation trial, ADHD-RS-IV clinician-rated symptom scores were significantly reduced (*P*≤.001) in all LDX groups (30, 50, and 70 mg/day [mg/d]) compared with placebo at weeks 1 to 4 and endpoint, and CGI-I ratings were significantly reduced at endpoint (*P*≤.001) [5]. A 12-month, open-label extension study enrolled eligible participants from the preceding short-term LDX trial. Results of the extension study demonstrated that ongoing LDX treatment was associated with a significant reduction in ADHD symptom scores from baseline of the prior study at all postbaseline visits and at endpoint (*P*<.001) [20]. In the current post hoc analysis, maintenance of clinical response and symptomatic remission during the 12-month open-label treatment was evaluated. The main goal of examining these post hoc analyses was to assess how useful these criteria are in providing additional clinically relevant information to evaluate the use of LDX in short- and long-term ADHD treatment.

## Methods

### Methods common to both clinical trials

Detailed methodology for both the short-term study and the follow-up, long-term extension study of LDX has been previously reported [5,20]. The studies were conducted from May to November 2006 and July 2006 to November 2007, respectively, at 44 sites in the United States, and were performed in accordance with the Declaration of Helsinki and the International Conference on Harmonization Guidelines for Good Clinical Practice. Institutional Review Boards at each site approved the protocol and conduct of the studies. All participants provided written informed consent. Both studies enrolled adults with a primary diagnosis of ADHD, based on the *DSM-IV-TR* criteria for the predominantly inattention subtype or the predominantly hyperactive/impulsive and combined subtypes.

For both studies, the primary outcome was change in ADHD-RS-IV with adult prompts total score from baseline at endpoint. ADHD-RS-IV scores were measured at each weekly visit in the short-term study [5], and at weekly and monthly visits during the extension study [20]. The ADHD-RS-IV contains 18 items corresponding to the criteria for an ADHD diagnosis described in the *DSM-IV-TR.* Secondary efficacy measures in both studies included the CGI-Severity (CGI-S) scale assessed at baseline of the short-term study (carried forward for the extension study), and the CGI-I scale assessed at all postbaseline visits in both studies. The CGI-S is used to rate the severity of symptoms on a 7-point scale ranging from 1 (normal, not at all ill) to 7 (among the most severely ill participants) at baseline. Symptom improvement was rated by the clinician on the CGI-I using a 7-point scale ranging from 1 (very much improved) to 7 (very much worse).

Safety assessments for both studies included spontaneously reported adverse events (AEs), vital signs, electrocardiograms (ECGs), routine clinical laboratory monitoring, and physical examinations (eg, height and weight), as previously reported [5,20]. Treatment-emergent AEs (TEAEs) referred to events with onset after the first date of treatment and no later than 3 days following termination of treatment.

### Short-term placebo-controlled study

In the 4-week, multicenter, placebo-controlled, double-blind, parallel-group, forced dose-escalation trial, [5] participants were randomly assigned in a 2:2:2:1 ratio to receive oral LDX (30, 50, or 70 mg/d) or placebo. Study phases included screening/washout, baseline randomization and measures, and a 4-week double-blind evaluation of LDX versus placebo. All participants initiated treatment with the 30-mg/d dose; participants assigned the higher doses initiated treatment with 30 mg/d, and the dose was increased in 20-mg increments, as assigned, at weekly intervals to 50 mg/d or 70 mg/d. Inclusion and exclusion criteria have been previously reported [5]. Briefly, key inclusion criteria included adults (≥18 to 55 years of age) diagnosed with ADHD according to *DSM-IV-TR* criteria with a baseline ADHD-RS-IV score ≥28. Exclusion criteria included individuals with comorbid psychiatric disorders; history of seizures, hypertension, tic disorder; Tourette disorder; pregnant or lactating women; positive urine drug result at screening or baseline; current medication use that might confound the results of the study or increase risk to the participant; clinically significant ECG; and any concurrent chronic or acute illness, or unstable medical condition.

### Open-label extension study

Participants enrolled in the short-term study for ≥2 weeks without AEs that would preclude continued treatment with LDX were eligible to participate in the long-term (12-month), open-label, single-arm study [5,20]. Baseline vital signs, ECG findings, and weight were carried over from the final visit of the previous study for participants enrolling within 7 days of finishing that study; otherwise, participants underwent a full screening and washout period with baseline assessments. Efficacy measures for all participants were carried forward from baseline of the prior trial. LDX treatment was initiated at 30 mg/d and, based on clinical judgment, could be increased or decreased in 20-mg increments at subsequent visits to achieve optimal efficacy and tolerability over a 4-week titration period. Adults were titrated to an “optimal” LDX dose (30 mg/d [n=44], 50 mg/d [n=112], and 70 mg/d [n=171]) over 4 weeks. Treatment was then maintained for up to 11 months during which time dosage could be adjusted up or down by 20-mg increments at monthly study visits, as deemed appropriate by the study investigator. The minimum dose was 30 mg/d while the maximum was 70 mg/d.

### Clinical response and symptomatic remission analyses

Table 1 summarizes post hoc analysis criteria for clinical response and symptomatic remission [9,12,18]. In the long-term trial, re-emergence of symptoms following clinical response or symptomatic remission was defined as failure to meet the criteria for response or remission at a later visit. Once having failed to meet such criteria, those participants were removed from further analysis. Maintenance of clinical response or symptomatic remission was evaluated among participants who met response/remission criteria and completed the 4-week dose-optimization phase in the long-term trial. It was decided that the study design of the short-term trial, because of the 4-week duration and the forced-dose titration schedule, was not suited for an analysis of maintenance of response or remission. Similarly, because the study design of the long-term trial did not require a defined washout period for those participants directly rolling over from the short-term study, there is a potential for carryover effect. Therefore, short-term data were used for the analysis of time to median clinical response or symptomatic remission, and long-term data were used for the analysis of time to median loss of clinical response or symptomatic remission.

**Table 1:** **Definitions and criteria for post hoc analysis of clinical response and symptomatic remission**[12,18]

**Terms**	**Definitions and Criteria**
**Clinical response**	≥30% reduction in ADHD-RS-IV with adult prompts total score^a^ and a CGI-I rating of 1 or 2
**Symptomatic remission**	ADHD-RS-IV total score ≤18 (average per-item score ≤1)
**Time to median clinical response or symptomatic remission**	Time by which half the original sample achieves criteria for clinical response or symptomatic remission
**Loss of clinical response or symptomatic remission status**	Failure to meet criteria for clinical response or symptomatic remission after having achieved that status at a previous visit
**Maintenance of clinical response or symptomatic remission at endpoint**	*4-week trial:*
	Participants meeting criteria for clinical response or symptomatic remission at postbaseline visits, enrolled at endpoint, and meeting criteria without interruption
	*Long-term trial:*
	Participants meeting criteria for clinical response or symptomatic remission at postbaseline visits and meeting the criteria without interruption up to endpoint

^a^Relative to baseline ADHD-RS-IV total score in the 4-week trial.

Abbreviations: ADHD-RS-IV = Attention-Deficit/Hyperactivity Disorder Rating Scale IV; CGI-I=Clinical Global Impressions-Improvement.

### Statistical analysis

Efficacy analyses were performed on the intention-to-treat population, also referred to as the efficacy population, defined in the 4-week study as all randomized and treated participants with a primary efficacy assessment at baseline and at least once postrandomization, and in the long-term study as those with at least one postbaseline primary efficacy assessment. Differences among all LDX dose groups in the 4-week study for time to median clinical response or symptomatic remission were analyzed post hoc with a log-rank statistic test. No adjustments for multiple comparisons were performed on the optimal-dose group for statistical comparisons.

Assessment of time to median first clinical response or symptomatic remission and of time to median first loss of clinical response or symptomatic remission was performed using survival analysis [21]. In the short-term study, percentages of participants achieving clinical response or symptomatic remission and time to median first clinical response or symptomatic remission from baseline were calculated for the efficacy population. In the long-term study, percentages of participants losing clinical response or symptomatic remission at any point during the maintenance phase of the study were calculated from the start date of the study. Only participants who achieved this clinical response or symptomatic remission status within the dose-titration phase and who continued into the maintenance phase of the study were included in this analysis. Survival analysis documents the first occurrence of an event and cannot take into consideration participants who lose clinical response or symptomatic remission at one time point but subsequently regain that status. Participants who discontinued the study or who reached study endpoint while in clinical response or symptomatic remission were censored. Loss of symptomatic remission status did not preclude continuing in the post hoc analysis as a clinical responder. Specifically, it is important to note that survival analyses conducted in this study were designed to only examine loss of clinical response and symptomatic remission, while censoring was defined in these analyses as removing a participant who did not have the event at their last study visit.

## Results

### Short-term study clinical response and symptomatic remission outcomes

A total of 420 participants were randomized; 414 participants were included in the efficacy population. Baseline demographic and clinical characteristics by treatment group (LDX 30, 50, or 70 mg/d or placebo) have been described in detail previously [5]. Briefly, mean (SD) baseline ranges for age were 34.2 (10.0) years to 35.8 (10.5) years, for weight were 173.1 (37.8) pounds to 181.3 (39.1) pounds, and for height were 67.4 (3.7) inches to 67.9 (3.9) inches and were comparable among the LDX and placebo treatment groups in the safety population. Most participants were Caucasian (77.4%-88.5%), and slightly more than half were men (51.6%-56.4%). In accordance with enrollment criteria, all participants exhibited ADHD symptoms at baseline that were at least moderate in severity (eg, CGI-S=4: 30%-44%, CGI-S >4: 56%-70%).

**Figure 1:**
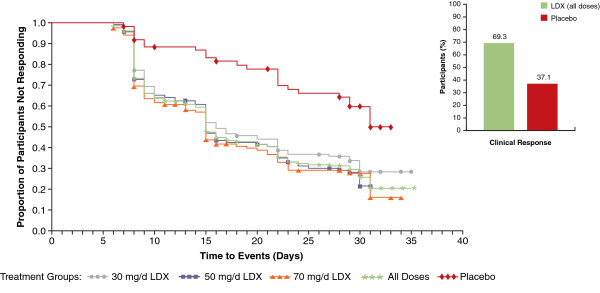
**Kaplan-Meier plot: time to median clinical response from baseline in the short-term study by LDX treatment group (efficacy population, n=414).** Log rank *P*-value: <.0001. *P*-value indicates overall significant effect among the treatment groups and was not specifically tested between any individual dose groups. Abbreviations: d=day; LDX=lisdexamfetamine dimesylate.

**Figure 2:**
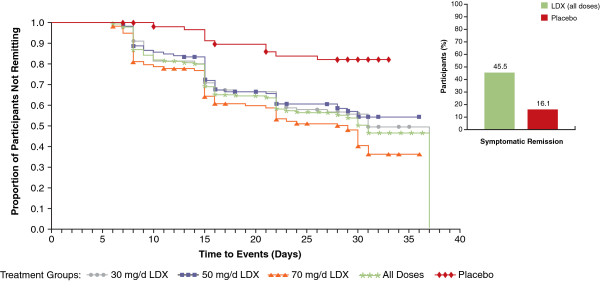
**Kaplan-Meier plot: time to median symptomatic remission from baseline in the short-term study by LDX treatment group (efficacy population, n=414).** Log rank *P*-value: <.0001. *P*-value indicates overall significant effect among the treatment groups and was not specifically tested between any individual dose groups. Abbreviations: d=day; LDX=lisdexamfetamine dimesylate.

Table 2 summarizes the proportions of participants who met criteria for clinical response and symptomatic remission at any time during the study and the time to median (95% confidence interval [CI]) clinical response and symptomatic remission from baseline by which these criteria were met. Of 352 participants treated with LDX (all doses combined), 244 (69.3%) responded to treatment (vs 23 of 62 [37.1%] in the placebo group); based on Kaplan-Meier analysis, time to median (95% CI) clinical response was 15.0 (15.0, 17.0) days for all LDX dosage groups at that point in time (overall log rank *P*<.0001) (Figure 1 and Table 2). Although the dosing was not the same over time, a total of 160 (45.5%) participants given LDX (all doses combined) achieved symptomatic remission (vs 10 of 62 [16.1%] in the placebo group); based on Kaplan-Meier analysis, time to median (95% CI) symptomatic remission was 31.0 (28.0, 37.0) days for all LDX doses (overall log rank *P*<.0001) (Figure 2 and Table 2).

**Table 2:** Clinical response and symptomatic remission outcomes in the short-term study by treatment group (efficacy population, n=414)

	**LDX**				**Placebo**
	**30 mg/d (n=115)**	**50 mg/d (n=117)**	**70 mg/d (n=120)**	**All Doses (n=352)**	**(n=62)**
**Clinical response, n (%)**	77 (67.0)	83 (70.9)	84 (70.0)	244 (69.3^**b**^)	23 (37.1^**b**^)
**Time to median clinical response,**^**a**^**d (95% CI)**	16.0 (15.0, 22.0)	15.0 (15.0, 21.0)	15.0 (13.0, 18.0)	15.0 (15.0, 17.0)	31.0 (29.0, NA)
**Symptomatic remission, n (%)**	51 (44.3)	47 (40.2)	62 (51.7)	160 (45.5^**c**^)	10 (16.1^**c**^)
**Time to median symptomatic remission, d (95% CI)**	31.0 (23.0, 37.0)	NA	29.0 (21.0, 31.0)	31.0 (28.0, 37.0)	

^a^Relative to baseline time to median clinical response in the 4-week trial.

^b^See Figure 1.

^c^See Figure 2.

Abbreviations: *d* day, *CI* confidence interval, *LDX* lisdexamfetamine dimesylate, *NA* not assessed.

### Long-term study clinical response and symptomatic remission outcomes

As previously reported, [20] the long-term open-label study enrolled 349 adults; 337 (96.6%) within 7 days of the end of the short-term study [5]. The efficacy population consisted of 345 participants (n=296, LDX; n=49, placebo, in the prior study), has been described previously [20] and was generally comparable to that in the short-term trial. Of the 345 participants in the efficacy population, 325 (94.2%) participants met criteria for clinical response at any point during the study, and 286 (82.9%) participants met criteria for symptomatic remission from baseline. Overall, 285 of 345 (82.6%) participants met criteria for clinical response and 227 of 345 (65.8%) participants met criteria for symptomatic remission at their final study visit (ie, end of study/or early termination [ET]).

Eighteen (5.2%) participants of the 345 in the efficacy population were excluded from the time to median events analysis in the maintenance phase because of discontinuations before the first maintenance-phase visit or lack of evaluable maintenance-phase efficacy data [22]. A total of 327 participants completed dose titration, entered the maintenance phase of the study, and had evaluable maintenance-phase data; 191 participants completed the trial.

**Figure 3:**
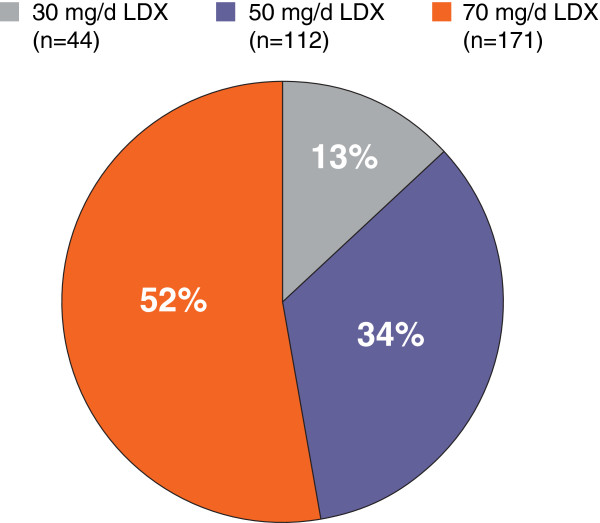
**Percentage of participants optimized to each dose, among participants who completed dose optimization (n=327).** Percentages have been rounded; total tally may not equal 100%. Abbreviations: d=day; LDX=lisdexamfetamine dimesylate.

Of the 327 participants who completed dose titration, 315 participants continued into the third month or thereafter and were evaluable for a subsequent change in dose. The percent of participants optimized to each LDX dose, among participants who completed dose optimization (n=327) are illustrated in Figure 3. It should be noted that the treatment dose at the end of dose titration was not necessarily the dose when clinical response or symptomatic remission was attained. Of the 148 participants titrated to either 30 mg/d (n=40) or 50 mg/d (n=108) LDX at the start of the maintenance phase, 54 (36.5%) participants had a dose increase. Sixty-three (22.9%) of 275 participants, titrated to either 50 mg/d (n=108) or 70 mg/d (n=167) LDX at the start of the dose-maintenance phase had a dose decrease; 113 participants had either an increase or a decrease from their titrated dose.

**Figure 4:**
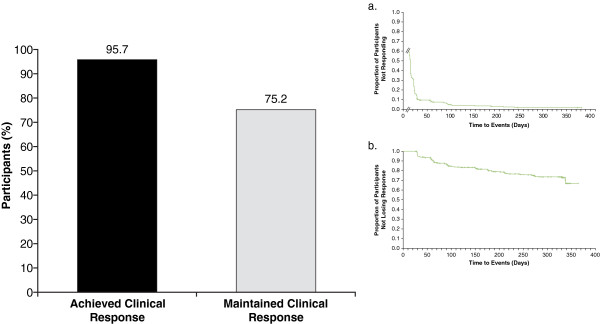
**Percentage of participants who achieved clinical response and maintained clinical response and Kaplan-Meier time course (inset) of attainment (a) and loss (b) of clinical response from baseline in the long-term study for all LDX treatment groups.** For attainment (**a**), log rank *P*-value: .0115; time to first clinical response was calculated from first day of study for participants who entered the maintenance phase of the study (n=327), response status at the start of the long-term study was not determined, and first on-treatment assessment of Attention-Deficit/Hyperactivity Disorder Rating Scale IV and Clinical Global Impressions-Improvement were at week 1; *P*-value indicates overall significant effect among the treatment groups and was not specifically tested between any individual dose groups. For loss (**b**), log rank *P*-value: .5531; time to loss of clinical response during the maintenance phase was calculated for participants who met criteria for response (n=278) at week 4 (visit 5); *P*-value indicates overall significant effect among the treatment groups and was not specifically tested between any individual dose groups. Abbreviation: LDX=lisdexamfetamine dimesylate.

**Figure 5:**
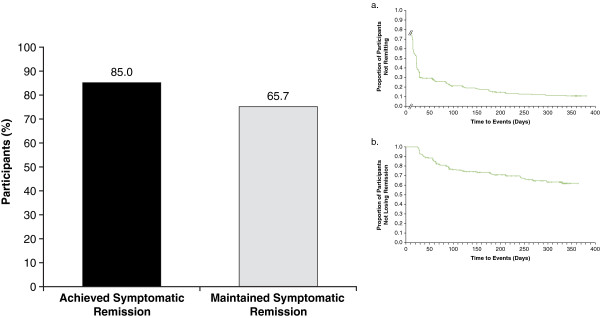
**Percentage of participants who achieved symptomatic remission and maintained symptomatic remission and Kaplan-Meier time course (inset) of attainment (a) and loss (b) of symptomatic remission in the long-term study for all LDX treatment groups.** For attainment (**a**), log rank *P*-value: .0012; time to first symptomatic remission was calculated from the first day of the study for participants who entered the maintenance phase of the study (n=327), remission status at the start of the long-term study was not determined, and first on-treatment assessment of Attention-Deficit/Hyperactivity Disorder Rating Scale IV was at week 1; *P*-value indicates overall significant effect among the treatment groups and was not specifically tested between any individual dose groups. For loss (**b**), log rank *P*-value: .0385; time to loss of symptomatic remission during the maintenance phase was calculated for participants who met criteria for remission (n=213) at week 4 (visit 5); *P*-value indicates overall significant effect among the treatment groups and was not specifically tested between any individual dose groups. Abbreviation: LDX=lisdexamfetamine dimesylate.

Of the 327 participants with evaluable maintenance-phase data, 313 (95.7%) met criteria for clinical response during the study with LDX (all doses) treatment (Table 3 and Figure 4). Moreover, 278 (85.0%) of these 327 participants met criteria for symptomatic remission during the study with LDX (all doses) treatment (Table 3 and Figure 5).

**Table 3:** Clinical response and symptomatic remission outcomes in the long-term study among participants who completed dose optimization, by optimized dose (n=327)

	**LDX**			
	**30 mg/d (n=44)**	**50 mg/d (n=112)**	**70 mg/d (n=171)**	**All Doses (n=327)**
**Clinical response, n (%)**	42 (95.5)	107 (95.5)	164 (95.9)	313 (95.7^a^)
**Symptomatic remission, n (%)**	38 (86.4)	95 (84.8)	145 (84.8)	278 (85.0^b^)

^a^See Figure 4.

^b^See Figure 5.

Abbreviation: *LDX* lisdexamfetamine dimesylate.

There were 278 participants who completed the dose-optimization phase and were classified as responders at entry to the maintenance phase; 209 (75.2%) participants did not experience a loss of clinical response during the maintenance phase or at each of their subsequent visits during the 12-month study (Figure 4). A total of 213 participants completed the dose-optimization phase and were classified as symptomatic remitters at entry to the maintenance phase. In addition, 140 (65.7%) participants, of which not all completed each study visit (ie, ET), did not experience a loss of symptomatic remission during the maintenance phase or at each of their subsequent visits during the 12-month study (Figure 5).

### Safety

Safety data were previously reported in detail for the short- and long-term studies [5,20]. In the short-term study, 5.9% of participants discontinued due to TEAEs in the LDX group, and 1.6% in the placebo group. In the long-term study, 8.0% of participants discontinued due to TEAEs. In the short-term study, TEAEs were reported by 79% of participants taking LDX and 58% taking placebo. In the long-term study, TEAEs were reported in 87.7% of participants. TEAEs reported by >10% of participants with LDX treatment in the short-term study included decreased appetite (27%), dry mouth (26%), headache (21%), and insomnia (19%) and in the long-term study included upper respiratory tract infection (22%), insomnia (20%), headache (17%), dry mouth (17%), decreased appetite (14%), and irritability (11%). In both trials, the majority of AEs were mild or moderate in severity.

## Discussion

Limited data exist on how to monitor and optimize treatment in adults with ADHD. We propose that a 30% reduction on the ADHD-RS-IV along with a 1- to 2-point improvement in CGI is a useful measure of initial clinical response. Even more importantly, a score of 18 or less on the ADHD-RS-IV (the 18 symptoms of ADHD are mild or less on average) appears to be a realistic goal for ADHD treatment in adults. These post hoc analyses of data from a short-term study [5] and a long-term study [20] have shown that LDX in adults with ADHD is associated with clinical response and symptomatic remission that persist over time for many participants. In the 4-week study, the majority of participants met post hoc criteria for clinical response and nearly half met criteria for symptomatic remission. In the 12-month study, nearly all dose-optimized participants (95.7%) met criteria for clinical response and many participants (85.0%) met criteria for symptomatic remission at least once during the trial. Of the participants who met clinical response criteria, 75.2% continued to meet criteria at every subsequent visit, and of those who met symptomatic remission criteria, nearly two-thirds (65.7%) continued to meet symptomatic remission criteria at all subsequent visits.

In adults with ADHD, there are not many recognized and clinically relevant measures that have been established. Similar to treatment guidelines for major depression that have emphasized the importance of treating to levels of symptomatic remission, describing criteria for clinical response and symptomatic remission in adults with ADHD may provide a useful, clinically relevant measure. This may be more meaningful to clinicians than are assessments of group average scores and population norms on symptom rating scales, most of which may not be applied in a clinical setting [18]. Describing clinical response and symptomatic remission provides clinicians with outcome measures that resemble clinical approaches to patient assessment and treatment [18]. Applying these criteria in clinical practice will offer a useful assessment measure with benchmarks for optimal treatment. Time to median clinical response and symptomatic remission informs both the participant and the clinician about when noticeable therapeutic effects could be expected to emerge for most patients, setting the stage for more timely recognition of a need for dose optimization or medication switching. In the short-term study, time to median clinical response with LDX was achieved by most participants in approximately 2 weeks. Due to the forced-dose escalation design of the 4-week study, however, it is not possible to differentiate the relative contributions of dose level versus time on treatment for achieving clinical response criteria.

A number of investigators have described clinical response and symptomatic remission rates for stimulant and nonstimulant pharmacotherapy in adults with ADHD. In previous trials of adults with ADHD, clinical response has been defined either as at least a 30% decline from baseline in symptom scores on a validated rating scale of adult ADHD symptom severity [6,23,24] or as very much improved or much improved on ratings of global improvement at endpoint [3,4,25]. Studies using variable dosing designs have been conducted with other long-acting psychostimulants (besides LDX) in adults with ADHD. Clinical response rates ranged from 48.5% to 95.1% [3,4,6,23-25]. No previous reports have explicitly described symptomatic remission in adults with ADHD. Adler et al. [25] described the proportions of “normal to mildly ill” adults with ADHD based on endpoint CGI-S ratings; 64.6% met those criteria in the 5-week, short-term double-blind treatment phase (fixed dose of d-MPH extended release). In the open-label extension phase, 90.0% who switched from placebo and 92.7% who continued on active treatment met those criteria. Jain and colleagues [4] described participants with “normalization rates…[of] 73.7%” based on Conners’ Adult ADHD Rating Scales–Self Report Index T-scores of <65, which indicates that the severity of impairment is below clinical threshold levels. In the current analysis, 85.0% of participants in the long-term trial met the criteria for symptomatic remission; in 65.7% of these participants, symptomatic remission persisted with continued treatment. Study design differences may account for the broad variability seen among these trial outcomes. Importantly, definitions of clinical response and symptomatic remission varied widely, which is understood to lead to different outcomes [12,15,18]. Neither the current trial nor prior investigations have conducted head-to-head treatment comparisons, precluding meaningful comparisons of clinical response and symptomatic remission rates between treatments.

The criteria for clinical response used in this post hoc analysis were more stringent than most existing reports because they required improvement as assessed by 2 separate measures: ADHD-RS-IV with adult prompts and the CGI-I. This double-measure criterion is not without precedent. It has been used previously in adults with ADHD in a trial of osmotic-release oral system MPH [3]. Most trials, however, have defined clinical response based on a single measure, such as Medori and colleagues [24]. Several factors argue against a single cutoff criterion. For example, recent research has shown that, in adults, a 25% to 30% change from baseline to endpoint in ADHD-RS-IV score or an absolute change of approximately 10 to 15 points corresponds to a change of one level in CGI-I rating in both adults and children, [14] and this may not be clinically meaningful. A percent reduction in ADHD-RS-IV total score alone is not sufficient because participants who are severely ill at baseline may still exhibit significant symptoms [12]. Addition of the CGI-I criterion of 1 or 2, as applied in the current study, prevents such participants from being defined as clinical responders. The criteria for symptomatic remission applied in the current study are consistent with some definitions previously proposed for symptomatic remission [18,19] and represent a substantial treatment-associated decrease of symptoms, but they do not preclude the ongoing presence of mild residual symptoms that are not disabling. Further studies are required to validate this concept of symptomatic remission and its relationship to daily functioning and ADHD diagnostic threshold.

The application of different criteria for clinical response and symptomatic remission could lead to different results [15]. These classifications of clinical response and symptomatic remission are based on a current symptom scale and do not purport to reflect functional outcomes or other measures that may be used to characterize clinical improvement or recovery in a disease state. Recent studies [26,27] have focused on looking beyond symptom assessment to a more comprehensive understanding of functional outcomes and the real-world impact of symptom severity and treatment-related improvements [27]. For example, a combined analysis of randomized participants from 4 different studies that pooled participants across treatment groups suggested that a reduction of approximately 20 points on the ADHD-RS was associated with pronounced functional outcome improvement (social and behavioral) on the Life Participation Scale [28]. This result corresponded to a 50% to 65% improvement in symptom severity levels and demonstrated improvement in functional outcome status. The analysis also indicated that a threshold of 40% to 45% improvement in symptom severity was needed to achieve clinically apparent functional improvement [28]. Diagnostic criteria for ADHD in adult patients and the criteria for clinical response and symptomatic remission in these post hoc analyses were based on those listed in the *DSM-IV-TR*. Although changes in symptom thresholds of the diagnostic criteria may occur with the expected adoption of the revised version, *DSM 5* in 2013, current considerations maintain the requirement for 6 symptoms of either subtype to diagnose ADHD in adults [29]. Pending implementation of potential changes, it may be interesting for future analyses to examine the impact of revised criteria for symptom presentation on clinically relevant assessments of clinical response and symptomatic remission.

### Limitations

There were several limitations of the current studies and analyses. This publication is derived from post hoc analyses rather than predefined study endpoints. Both the short- and the long-term investigations excluded adult participants with common medical and psychiatric comorbidities; hence, current findings may not generalize to a broader clinical population. The use of a forced-dose escalation design in the short-term study may have led to nonrandom participant discontinuation (eg, possible poor tolerability in higher dose groups and lack of efficacy in lower dose groups). As in other trials that use open-ended questioning and spontaneous report to collect data on AEs, incidence may be underreported. In interpreting the findings on maintenance of response and remission in the long-term study, one should keep in mind that dose changes were possible for each individual during the maintenance phase of the study and, as such, may have contributed to maintenance of response and remission. The long-term study was open label, introducing the potential for investigator and participant bias toward reporting clinical improvement and, as with other long-term studies, participants leave over the course of the study for various reasons.

The criteria for response and remission were based on assessment of global and individual symptom level and severity. The impact of treatment on other important facets of ADHD, such as quality of life, or functional recovery/remission cannot be addressed. Although analyses evaluating participants who did not respond to treatment or who failed to maintain clinical response to treatment would be quite interesting and informative, it is beyond the scope of the current analysis. Also, not all participants respond to psychostimulant treatments, including LDX. Future work could examine characteristics and/or predictors of clinical nonresponse.

A limitation in interpreting time to occurrence of a specific event (in this study the event is defined as either loss of response or remission status) using the Kaplan-Meier survival analysis approach involves the process of censoring (removing) participants from the analysis. Participants are censored (removed) from the analysis if they have not had the event (eg, achieved response or remission status), and are no longer available to observe for the event. This occurred at the end of the study period, and when participants discontinued or were lost to follow-up while still a responders/remitters at their last visit. Therefore when we report that of 278 participants experiencing a clinical response on entering the maintenance phase of the study; 209 did not lose that response during their participation, not all had completed the study.

## Conclusions

The majority of adults receiving LDX achieved clinical response (ADHD-RS-IV total score reduction of ≥30% and a CGI-I rating of 1 or 2) within 15 days of initiating therapy during the short-term trial. The majority of participants (85%) met the criterion for symptomatic remission (ADHD-RS-IV total score of ≤18%) during the long-term, open-label, dose-optimization trial, and more than half maintained remitted status throughout continued LDX treatment. In order to confirm these results, prospective evaluation of clinical response and symptomatic remission endpoints is needed in future investigations. The safety profile of LDX was consistent with that of other long-acting psychostimulants.

## Abbreviations

ADHD-RS-IV: ADHD Rating Scale IV; AEs: Adverse events; ADHD: Attention-deficit/hyperactivity disorder; CGI-I: Clinical Global Impressions-Improvement; CGI-S: Clinical Global Impressions-Severity; CI: Confidence interval; d: day; *DSM-IV-TR*: *Diagnostic and Statistical Manual of Mental Disorders, Fourth Edition, Text Revision*; *DSM-5*: *Diagnostic and Statistical Manual of Mental Disorders, Fifth Version*; ET: Early termination; ECGs: Electrocardiograms; d-MPH: dexmethylphenidate; LDX: Lisdexamfetamine dimesylate; MPH: Methylphenidate; NA: Not assessed; SNAP-IV: Swanson: Nolan, and Pelham, Version IV; TEAEs: Treatment-emergent AEs.

## Competing interests

Dr Mattingly serves/d as a consultant for GlaxoSmithKline, Johnson & Johnson, Novartis, Sepracor, Shionogi, and Shire; receives/d research support from Sepracor; receives/d honoraria from Forest, GlaxoSmithKline, Johnson & Johnson, Lilly, Novartis, and Shire; serves/d as speaker or advisory board member for Sepracor.

Dr Richard Weisler, in his career, has been a consultant to, on the Speaker’s Bureaus of, and/or received research support from the following: Abbott - Speaker’s Bureau, Consultant, Received Research Support, Agency for Toxic Substances and Disease Registry- Consultant, AstraZeneca - Speaker’s Bureau, Consultant, Received Research Support, Biovail - Speaker’s Bureau, Consultant, Received Research Support, Bristol-Myers Squibb - Speaker’s Bureau, Consultant, Received Research Support, Stockholder has held or holds stock, Burroughs Wellcome - Speaker’s Bureau, Received Research Support, Cenerx - Received Research Support, Centers of Disease Control and Prevention - Consultant, Cephalon - Speaker’s Bureau, Consultant, Received Research Support, Ciba Geigy - Speaker’s Bureau, Received Research Support, CoMentis - Received Research Support, Corcept - Consultant, Cortex - Stockholder has held or holds stock, Dainippon Sumitomo Pharma America - Received Research Support, Eisai - Received Research Support, Eli Lilly - Speaker’s Bureau, Consultant, Received Research Support, Forest - Speaker’s Bureau, Consultant, Received Research Support, GlaxoSmithKline - Speaker’s Bureau, Consultant, Received Research Support, Janssen Speaker’s Bureau, Received Research Support, Johnson & Johnson - Speaker’s Bureau, Consultant, Received Research Support, Lundbeck - Received Research Support, McNeil Pharmaceuticals - Received Research Support, Medicinova - Received Research Support, Medscape Advisory Board - Consultant, Merck - Received Research Support, Stockholder has held or holds stock, National Institute of Mental Health –Consultant, Received Research Support, Neurochem - Received Research Support, New River Pharmaceuticals - Received Research Support, Novartis - Speaker’s Bureau, Received Research Support, Organon - Speaker’s Bureau, Consultant, Received Research Support, Otsuka America Pharma - Consultant, Pfizer - Speaker’s Bureau, Consultant, Received Research Support, Stockholder has held or holds stock, Pharmacia - Consultant, Received Research Support, Repligen - Received Research Support, Saegis - Received Research Support, Sandoz - Received Research Support, Sanofi - Speaker’s Bureau, Consultant, Received Research Support, Sanofi-Synthelabo - Speaker’s Bureau, Consultant, Received Research Support, Schwabe/Ingenix - Received Research Support, Sepracor - Received Research Support, Shire - Speaker’s Bureau, Consultant, Received Research Support, Solvay - Speaker’s Bureau, Consultant, Sunovion – Speaker’s Bureau, Consultant, Received Research Support, Synaptic - Received Research Support, Takeda - Received Research Support, TAP - Received Research Support, Transcept Pharma - Consultant, Received Research Support, TransTech - Consultant, UCB Pharma - Received Research Support, Validus - Speaker’s Bureau, Consultant, Vela - Received Research Support, and Wyeth - Speaker’s Bureau, Consultant, Received Research Support.

Dr Young receives/d grant/research support from Cyberonics, Lilly, Novartis, Pfizer, Otsuka, and Shire; is/has been a speaker for AstraZeneca, Bristol-Myers, Cephalon, Forest, GSK, Lilly, McNeil, Novartis, Pfizer, Sepracor, Schering Plough, Shionogi, and Shire.

Mr Adeyi is an employee of Shire and holds stock and/or stock options in Shire.

Dr Dirks is an employee of Shire and holds stock and/or stock options in Shire.

Dr Babcock is an employee of Shire and holds stock and/or stock options in Shire.

Dr Lasser was an employee of Shire with stocks and stock options from 2008 to July 2012. From July 2012 to the present, he is an employee of Pharmanet/i3, an inVentiv Health company (no stock or options in Shire or any other company).

Dr Scheckner is an employee of Shire and holds stock and/or stock options in Shire.

Dr Goodman receives/d research support from Lilly and Company, McNeil, and Shire Inc.; receives/has received honoraria from McNeil, Shire Inc.; is/has been a speaker for American Professional Society of ADHD and Related Disorders, Audio-Digest Foundation, CME Inc, Medscape, SynerMed Communications, Temple University, Veritas Institute, WebMD; is/has been a consultant for Avacat, Clinical Global Advisors, McNeil, New River Pharmaceuticals, Novartis, Schering-Plough, Shire Inc., Major League Baseball, and Thompson Reuters; receives royalties from MBL Communications, Inc.

## Authors’ contributions

GM was an investigator on the parent study and participated in data acquisition, analysis, interpretation, and presentation. GM was fully involved in drafting the manuscript and revising the intellectual content of this manuscript. He has given final approval of this version. RW was an investigator on the parent study and participated in data acquisition, analysis, interpretation, and presentation. RW was fully involved in drafting the manuscript and revising the intellectual content of this manuscript. He has given final approval of this version. JY was an investigator on the parent study and participated in data acquisition, analysis, interpretation, and presentation. JY was fully involved in drafting the manuscript and revising the intellectual content of this manuscript. He has given final approval of this version. BA was a statistician involved in all post hoc data analysis, interpretation, and presentation. He was fully involved in drafting and revising the intellectual content of this manuscript. He has given final approval to this version. BD was the Director, Clinical Development and Medical Affairs, for this study and made substantial contributions to the analysis and interpretation of the data. He was deeply involved in drafting the manuscript and revising the intellectual content. He has given final approval of this version. TB was the Associate Director Scientific Publications, Clinical Development and Medical Affairs, for this study and made substantial contributions to the analysis and interpretation of the data. He was deeply involved in drafting the manuscript and revising the intellectual content. He has given final approval of this version. RL was the Senior Director, Clinical Development and Medical Affairs, for this study and made substantial contributions to the analysis and interpretation of the data. He was deeply involved in drafting the manuscript and revising the intellectual content. He has given final approval of this version. BS was the Director-Scientific Publications, Clinical Development and Medical Affairs, for this study and made substantial contributions to the analysis and interpretation of the data. He was deeply involved in drafting the manuscript and revising the intellectual content. He has given final approval of this version. DG was an investigator on the parent study and participated in data acquisition, analysis, interpretation, and presentation. JY was fully involved in drafting the manuscript and revising the intellectual content of this manuscript. He has given final approval of this version. All authors read and approved the final manuscript.

Presented at the 162nd Annual Meeting of the American Psychiatric Association; May 16–21, 2009; San Francisco, CA

Presented at the US Psychiatric and Mental Health Congress Conference and Exhibition; November 2–5, 2009; Las Vegas, NV

## Source of financial material and support

Clinical research was funded by the sponsor, Shire Development LLC, Wayne, PA, USA. Under the direction of the authors, Huda Abdullah, PhD, a former employee, and Michael Pucci, PhD, an employee of SCI Scientific Communication & Information (SCI), provided writing assistance for this publication. Editorial assistance in formatting, proofreading, copy editing, and fact checking was also provided by SCI. Shire Development LLC provided funding to SCI for support in writing and editing this manuscript. Although the sponsor was involved in the design, collection, analysis, interpretation, and fact checking of information, the content of this manuscript, the ultimate interpretation, and the decision to submit it for publication in *BMC Psychiatry* were made by the authors independently.

## Pre-publication history

The pre-publication history for this paper can be accessed here:

http://www.biomedcentral.com/1471-244X/13/39/prepub
